# Progress in Psychiatry

**Published:** 1899-12-30

**Authors:** 


### PROGRESS IN PSYCHIATRY.

(*Continued from page 49.*)

**Modern Problems.**—The growth of knowledge and of medical and scientific opinion during the closing decade of the century with regard to the problems of insanity may be broadly regarded in a threefold aspect. There is, first and foremost, the important practical question of the prevention of disease, and this, in reference to the insanities, means the gradual prevention of the preventable insanities. Secondly, we are met with the serious economical question in relation to a large proportion of patients who are the subjects of chronic insanities and allied infirmities of a hopeless and incurable nature, viz., the classification, housing, and management of these on a medico-economic basis. Thirdly, there arises for consideration the question of special provision—on the "hospital" system—for the treatment and cure of the curable insanities. Separate consideration is needful for each of these three aspects of the lunacy problem. The causes of insanity are most suitably surveyed from the standpoint adopted by Dr. Mercier,[1] who classifies them under the headings of heredity and stress. Causes operating under one or other or both of these two categories may result in bringing about various forms of mental degeneracy and disease. It will be seen that while some are more potent than others as factors of mental disease, not a few are capable of being limited and controlled, and are thus in a measure partially preventable. The consideration of this belongs to the branch of preventive medicine and ætiology.

**Ætiology and Morbid Heredity.**—In individual cases it is at times difficult to decide whether heredity or stress has played the chief part in determining the mental degeneracy of the patient. Statistics furnish some aid in determining the causes, but they must be interpreted with great caution. Different causes are so often intermingled in a given case that it is almost impossible to assign to any single cause this or that effect. And one of the best proofs of this is that nearly all ætiological classifications of insanity have failed, as a rule, to account for all the actual clinical facts and pathological lesions observed in insane patients, or to give a satisfactory classification for practical purposes. For the ætiology is complex, and moreover, the tracing and following out of the ætiological factors is beset with difficulty. Patients and friends sometimes will deliberately deny the existence of alcoholism, of suicidal impulses and propensities, and of actual attacks of insanity in the antecedents of the patient. A really satisfactory genealogical table—of the morbid heredity manifested both by antecedents and collaterals—is hard to obtain.

The causes of insanity include everything that is inimical to a healthy and well-balanced life—physical, moral, and social. Thus, whilst civilisation promotes mental and physical evolution, its demands on brain and mind are correspondingly augmented. A large number of individuals are thus found to be unfit for the harder trials and more strenuous stresses of life, and they therefore succumb in that portion of their organisation which feels the stresses most, viz., the brain and nervous system. On the other hand, the benefits conferred by civilisation, viz., increased comfort and improved hygiene, tend towards counteracting the ill effects of the stresses alluded to above. And on the whole a balance has to be struck between these two extremes—the good and the bad results—before it can be decided in individual cases that insanity is the net result of more than one cause.

(*a*) Deficient food and clothing constitute an important factor. The demands of daily labour impose on the organism the necessity of recuperation during the period of cessation from toil which constitutes sleep. Deficient food brings about anæmia, and it will be readily seen how this affects the nervous system, and more particularly the grey matter of the brain. For the nervous system, in proportion to its wants, is more abundantly supplied with blood than the bulk of the body tissues (muscular and connective tissues), and the grey matter of the brain has a blood supply which is three times as abundant as that of the white matter. Hence anæmia is, for the brain, a serious danger to its healthy working. It will also be seen that the conditions which favour anæmia, viz., deficient food and clothing, are but part of a wider group of conditions which include other evils, such as unhealthy dwellings and overcrowding. Moreover, we find a host of other noxious conditions associated with these which will be referred to in their proper place.

(*b*) Alcoholism is a factor of supreme importance in the causation of insanity and of various other forms of cerebral degeneration. Its mode of action is manifold. The intoxication of married couples on the wedding night has been shown by careful observers to result in the mental degeneracy of offspring conceived during the stage of intoxication. The noxious effect of the alcohol circulating in the blood, and thereby affecting the ova and spermatozoa, has been noted by observers, mainly of the French school (Morel, Combemale, Magnan, Féré), and has given rise to the adage, "an embrace given in a moment of intoxication may be fatal to an entire generation." The interesting experiments of Dr. Féré, which have been quoted by Dr. Lloyd Andriezen in a discussion on this subject at the last meeting of the British Medical Association,[2] further bear out this view. Chronic alcoholism indulged in for years is another and still more potent cause of degeneracy, both in the individual and in the offspring. Two results may be met with in the offspring of such parents, viz., a similarity of heredity (hereditary drink craving) or a tendency to the development of such neuroses as grave hysteria, epilepsy, imbecility and idiocy, and other forms of cerebral disease and insanity. It must be clearly comprehended that what is transmitted to the offspring is a diseased or debilitated protoplasm (ovum and spermatozoon), from which the tissues and organs have to be developed. In some cases the noxious effect may be so potent as to bring about death of the fœtus, followed by abortion, resulting from the action of alcohol during pregnancy. The varieties of morbid phenomena thus brought about in the offspring may range from premature death of the fœtus to meningitis, epilepsy, and suicidal tendencies. Morel[3] has given us in tabular form a genealogical table of the various diseases and morbid conditions which may result from chronic alcoholism in parents and offspring unto the fourth generation. It is as follows:—

1st generation: Alcohol excess, depravity, and moral degradation.
2nd generation: Hereditary drink craving, mania, general paralysis.
3rd generation: Hypochondriacal tendencies, melancholia and suicidal tendencies, homicidal tendencies.
4th generation: Weakmindedness and imbecility, sterile idiocy, and consequent extinction of the family.

Valuable collections of data illustrating the evil effects mentioned in the above general genealogical table will be found in the works of Dejerine[4] and Legraine.[5] It may be mentioned *en passant* that the habit of indulging in such intoxicating drinks as absinthe tends to produce somewhat similar effects to those of alcohol, with the exception that attacks of maniacal phrenzy and of epileptic insanity are peculiarly prone to result from the habit of absinthe drinking, a vice which prevails to a considerable extent in France.

(*c*) The incidence of syphilis, both on the military and the civil population, and the results of its ravages on the nervous system, can no longer be doubted. The lesions resulting from syphilis are manifold, and not the least among them are the syphilitic affections of the nervous system. As is the case with alcohol, so with syphilis, the pathological results may make their appearance, not only in the individual, but also in the offspring. The effects of acquired syphilis may appear in the individual in the form of syphilitic and parasyphilitic diseases of the brain and nervous system, not to mention other organs of the body which may also be affected. Among the syphilitic diseases proper are the encephalopathies of syphilitic arteritis, gummata and diffuse meningitis, and cerebral and spinal neoplasms of syphilitic origin. The parasyphilitic diseases include a group of more obscure diseases, supervening, as a rule, late, and as a remote consequent of the action of the syphilitic toxins on the nerve cells. Among these may be classed some cases of tabes dorsalis (locomotor ataxy) and of meningo-encephalitis (general paralysis of the insane), as taught by Fournier and others.[6] The cerebral and nervous degenerations of congenital syphilis may result in a variety of conditions. Among the more frequent are premature death of the fœtus and miscarriage, infantile convulsions and meningitis, paralytic, microcephalic, and other forms of idiocy, imbecility, or weakmindedness, and infantile and juvenile general paralysis (Alzheimer, Thiry).

(*d*) Tuberculosis, like syphilis, is capable of producing a cachexia in the offspring which may result in various forms of cerebral and mental degeneracy. The commoner results are idiocy and imbecility in their various forms, but most frequently in association with the pulmonary glandular and osseous affections of tuberculosis. The subjects of such affections are, as a rule, short-lived, seldom attaining to adult age.

(*e*) The neuropathic diathesis plays an important part in the evolution of insanity. The ideally healthy man does not become insane in the presence and under the stress of causes which are sufficient to develop insanity in his neuropathic fellow-man. This neuropathic predisposition or facility to succumb pathologically is often a subtle condition and difficult to define. It nevertheless exists. Of two men suffering from the same type of infectious fever (say, influenza or pneumonia), the one may develop a violent delirium, with complete mental confusion, and even with hallucinations of the senses, while the other scarcely exhibits any tokens of serious cerebral disturbance. Of two individuals taking equivalent doses of alcohol, the one may break out into violence and phrenzy with complete loss of self-control (acute alcoholic phrenzy), whereas the other exhibits only a mild exaltation and a genial feeling of *bien être*. Of two chronic heavy drinkers, one may eventually deteriorate into a condition of alcoholic insanity with delusions of suspicion and mental and moral failure, ending in alcoholic dementia and death in an asylum, the other may remain a hale and comparatively healthy toper and live a relatively respectable life to old age. Again, two girls may be the victims of seduction and desertion, and one may become insane and suffer from melancholia and make repeated attempts at self-destruction, whereas the other may rapidly recover from her shame and sorrow without manifesting any actual mental alienation. Finally, of two pregnant women going through the stresses of parturition and the puerperium under similar conditions, the one may develop an acute delirium with insane excitement and hallucinations (acute puerperal mental confusion), whereas the other passes through a little restlessness and irritability and a mild pyrexia lasting only two or three days, and with no untoward after-effect. Such facts of everyday experience show that a fundamental difference exists, viz., a neuropathic predisposition in the one case which facilitates the development of insanity. To certain of the clinical symptom-complexes of this morbid predisposition the terms "nervousness," "neurasthenia," and "neurosis" have been applied. Of one of these, viz., neurasthenia, it may at any rate be said that it has an individuality of its own which entitles it to rank with epilepsy, hypochondriasis, hysteria, &c., as a distinct ailment. "It is a mistake to suppose," says Möbius,[7] "that the majority of the descendants of the insane escape nervous affections altogether. The more carefully the members of a neuropathic family are scrutinised, the more frequently does it appear that the apparently healthy members frequently exhibit the mental and bodily stigmata of degeneration. Such people are grievous sufferers from the so-called trivial neuroses which, though they may seem to be of little importance, yet cause throughout the lifetime of these persons an amount of suffering which requires consideration." Among other manifestations of the neuropathic taint may be mentioned incorrigible tendencies to vagrancy and prostitution, suicidal impulses, and youthful sexual and moral perversions.

Mercier: Sanity and Insanity; Contemporary Science Series, London 1890. [2] Brit. Med. Jour., Section of Psychology, Sept. 1899. [3] Traite des Degenerescences, 1860. [4] L'heredite dans les Maladies du Systeme nerveux, Paris, 1886. [5] Alcoolisme et Degenerescence Sociale, Paris, 1890. [6] Les Affections Parasyphilitiques, Paris, 1894. [7] Uber nervösen Familien, 1884.

